# Research Progress and Applications of Bovine Genome in the Tribe *Bovini*

**DOI:** 10.3390/genes15040509

**Published:** 2024-04-18

**Authors:** Xingjie Du, Yu Sun, Tong Fu, Tengyun Gao, Tianliu Zhang

**Affiliations:** 1 College of Animal Science and Technology, Henan Agricultural University, Zhengzhou 450046, China; 17613064845@163.com (X.D.); sunyu95@163.com (Y.S.); futong2004@126.com (T.F.); dairyfarm@163.com (T.G.); 2 Henan International Joint Laboratory of Nutrition Regulation and Ecological Raising of Domestic Animal, College of Animal Science and Technology, Henan Agricultural University, Zhengzhou 450046, China

**Keywords:** genomics, genome annotation, functional genomics, adaptive evolution, database

## Abstract

Various bovine species have been domesticated and bred for thousands of years, and they provide adequate animal-derived products, including meat, milk, and leather, to meet human requirements. Despite the review studies on economic traits in cattle, the genetic basis of traits has only been partially explained by phenotype and pedigree breeding methods, due to the complexity of genomic regulation during animal development and growth. With the advent of next-generation sequencing technology, genomics projects, such as the 1000 Bull Genomes Project, Functional Annotation of Animal Genomes project, and Bovine Pangenome Consortium, have advanced bovine genomic research. These large-scale genomics projects gave us a comprehensive concept, technology, and public resources. In this review, we summarize the genomics research progress of the main bovine species during the past decade, including cattle (*Bos taurus*), yak (*Bos grunniens*), water buffalo (*Bubalus bubalis*), zebu (*Bos indicus*), and gayal (*Bos frontalis*). We mainly discuss the development of genome sequencing and functional annotation, focusing on how genomic analysis reveals genetic variation and its impact on phenotypes in several bovine species.

## 1. Introduction

The domestication and breeding of the *Bovini* tribe (taurine cattle, yak, buffalo, zebu, and gayal) have made an indispensable contribution to the development of human civilization since the early Holocene. Domesticated cattle, raised in various climates and production conditions in the world, provide meat, milk, hides, and other essential products for humans. The yak is crucial in the high-altitude regions of the Tibetan Plateau and surrounding areas. Yaks adapt well to extreme environments [1], characterized by low oxygen levels, harsh climate, and limited food resources, which equip them with developed cardiopulmonary function [2], enhanced foraging ability [3], and high energy metabolism [4]. The buffalo, praised by the Food and Agriculture Organization of the United Nations, is the primary livestock that feeds the world’s population. Domesticated water buffaloes are mainly distributed in Asia, accounting for 97% of the population, and are further divided into the swamp buffalo (2n = 48) and the river buffalo (2n = 50) [5]. Swamp buffaloes are characterized by docility and robust endurance, and provide labor for traditional agricultural rice cultivation. River buffalo cattle are prized for their high-quality milk, typically characterized by lower cholesterol but higher caloric and fat content than cow’s milk [6]. In addition, the zebu, also known as indicine cattle or humped cattle, is native to the Indian subcontinent and distributed in various tropical regions. One of the most prominent features of zebu cattle is the hump on their shoulders and neck, which distinguishes them from other cattle breeds. Zebu cattle are well adapted to tropical regions, especially semi-arid environmental conditions, with resistance to heat, parasites and infectious diseases [7]. The gayal, also named the mithan, mithun, or Drung cattle, is a rare semi-wild bovine species that mainly inhabits the hill forest areas of India, China, Bangladesh, Myanmar, and Bhutan [8]. Significant physiological advantages of gayal include well-developed heart and lungs, strong digestion ability, and tender meat. These distinctive characteristics make them valuable resources in the livestock farming industry.

**Figure 1:**
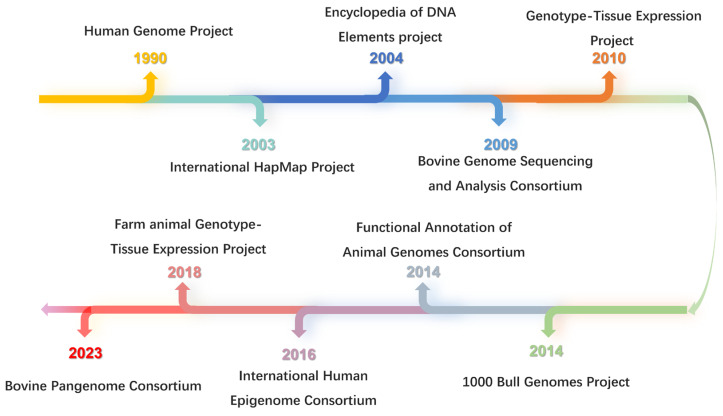
Progress in human and bovine genome projects.

Genomics studies will help us to understand the formation and genetic mechanisms of distinct traits in several bovine species. Inspired and guided by the Human Genome Project technology and methodology [9]; research projects including HAPMAP [10], the Encyclopedia of DNA Elements (ENCODE) project [11], and the Genotype-Tissue Expression (GTEx) project [12]; and numerous international consortia including the Bovine Genome Sequencing and Analysis Consortium [13], the Bovine HapMap Consortium [14], the 1000 Bull Genomes Project [15], the Bovine Genome Variation Database [16], and the Bovine Pangenome Consortium [17], coordinates were initiated to study functional genomics in cattle (Figure 1). Meanwhile, to characterize the functional element maps of domesticated animal genomes, including cattle, the Functional Annotation of Animal Genomes (FAANG) consortium was proposed to understand the genotype-to-phenotype link in farm animals [18]. Furthermore, the FarmGTEx project provided a public resource for regulatory variant discovery and molecular phenotype prediction in domesticated animals [19]. These fundamental works in the field of bovine genomics are the theoretical basis for functional genomics research, and breed germplasm resource innovation.

Up to now, the genome assembly and annotation of several bovine species have been completed. The first to complete the genome sequence determination was Heifer cattle (*B. taurus*), followed by zebu (*B. indicus*) [20], yak (*B. grunniens*) [21,22], water buffalo (*B. bubalis*) [23], gayal (*B. frontalis*) [24], etc. (Table 1). Furthermore, based on these reference genomes, critical genetic analyses have been conducted using high-throughput sequencing technologies such as genomic resequencing, transcriptomics, and epigenomics. We reviewed all publications including the keywords “Bovine”, “Genome annotation”, “Functional genomics”, “Adaptive evolution”, and “Bovine Database” in the PubMed database (https://www.ncbi.nlm.nih.gov/pubmed, accessed on: 1 January 2024). This review focuses on several bovine species and summarizes the reference genome assembly and annotation, functional genomics, and adaptive evolution of cattle. It also provides an outlook on the research trends in genomics, aiming to lay the foundation for the genetic analysis of future economic traits in cattle.

## 2. Development of the Tribe *Bovini* Genome

The assembly and annotation of bovine reference genomes are essential to performing genetic and genomic analyses. In 2009, the first genome sequence of taurine cattle was sequenced and assembled [13]. The estimated genome size was 2.87 Gb and contained a minimum of 22,000 protein-coding genes. Segmental duplications and positive selection analysis identified immune-related genes (e.g., *IFNAR2*, *IL23R*, *IL24*, *IL15*, *LEAP2*). To improve the continuity of the bovine genome, a high-quality reference genome was constructed in combination with modern technologies such as single-molecule sequencing. The genome length was 2.72 Gb, with contig N50 and scaffold N50 improved by 323 and 83 times, respectively [25]. Subsequently, the genomes of multiple breeds (Fleckvieh-Simmental and Charolais) were sequenced and assembled separately [26,27]. The ARS_Simm1.0 was similar in length to the ARS-UCD1.3 at 2.86 Gb. The distinctive genetic characteristics of yaks make them an interesting subject for genome research. In 2012, the first draft genome of yak was released [21]. The total genome size was 2.65 Gb, and nearly 99.6% of the sequences were consistent with the cattle (*Bos taurus*) genome (UMD3.1). Comparative genomic analysis identified candidate genes involved in sensory perception (*GPCR*), hypoxia response (*ADAM17*, *ARG2*), and nutrient metabolism (*CAMK2B*, *GCNT3*, *HSD17B12*, *WHSC1*, and *GLUL*). More complete and accurate genomes of wild and domestic yaks were constructed based on multiple sequencing strategies. The assembled genome was slightly smaller than previously reported, but had a similar number of protein-coding genes (22,931 and 23,143 protein-coding genes in the wild and domestic yak genomes, respectively) (Table 1).

Extensive research into buffalo genomics has benefited from advances in high-throughput sequencing technology. In 2017, the river buffalo genome reference sequence was released, and the genome sequence was 2.83 Gb in size [30]. Subsequently, a chromosome-level assembly of the water buffalo genome was created using single-molecule sequencing and Hi-C data [23]. Compared to the previous short read-based buffalo genome, this genome improves the contig N50 more than a thousand-fold, and helps annotate gene clusters, such as major histocompatibility complexes (MHCs) [23]. Recently, to further understand the different domestication characteristics of indicine breed genomes, the first genome from a Nellore breed was sequenced and de novo assembled using the ABI SOLiD sequencing platform [20]. Compared to the cattle (*B. taurus*) genome, the Nellore genome was highly parallel at the nucleotide level of all autosomes and X chromosomes [37]. Then, the draft genome assemblies of four *B. indicus* breeds were constructed. In addition, complex allelic variations hinder the assembly of haplotype sequences in diploid genomes. A diploid assembly of an outbred F1 hybrid between Angus (*B. taurus*) and Brahman (*B. indicus*) was completed using a trio binning strategy [38]. The F1 haplotype genome with a haploid NG50 in Angus and Brahman was 26.6 Mb and 23.3 Mb, respectively, surpassing the quality of previous *B. taurus* and *B. indicus* reference genomes. Studying the gayal genome contributes to understanding the mechanisms of its environmental adaptation and chromosome fusion. The first genome from a female Chinese gayal had been de novo assembled and annotated using the Illumina genomic sequencing platform [34]. The assembled genome size was about 2.85 Gb. Subsequently, an adult female Mithun from India was built using a hybrid assembly strategy [35]. The final genome size was about 3.0 Gb. Gene annotation identified 26,884 protein-coding genes. To characterize the mechanisms underlying a chromosome fusion event, a male Drung cattle genome was de novo assembled [24] and 29 scaffolds were identified, of which one scaffold sequence (Fragscaffold60) had the presence of cattle satellite I with 28 tandem repeats. Furthermore, a chromosome-level genome for gayal has been completed [32]. This genome sequence provides base pair-level resolution for Robertson translocations (2; 28) and reveals interactions between chromosomal 2 and 28.

## 3. Bovine Pangenome Studies

To obtain broader and more representative genomic information across bovine species, pangenome has gradually become a new strategy for genomics research. Crysnanto et al. [39] constructed a bovine pangenome with six reference genomes across three bovine species, including yak (*B. grunniens*), taurine (*B. taurus*), and Brahman (*Bos taurus indicus*) cattle. The bovine pangenome contained 70,329,827 non-reference bases. Non-reference sequences were annotated using transcriptomes, primarily encoding proteins involved in immune response and pathogen-mediated immunomodulation. Leonard et al. [40] constructed a structural variant (SV)-based pangenome from the offspring of three bovine trios. Pangenome topology analysis identified 90,000 SVs, including variants affecting *TAS2R46*, *QRICH2*, *PRDM9*, and *HSPA1A*, that may be functionally associated with reproduction and dietary habitat adaptation. Subsequently, Zhou et al. [41] built a pangenome covering 57 breeds, and identified 83 Mb of novel sequence. Genetic structural analysis detected novel structural variations, including deletion, duplication, and inversion variants, which provided genetic information about loci in the bovine genome that may underlie the phenotypic diversity. Dai et al. [42] constructed a Chinese indicine pangenome across southern China, and identified 148.5 Mb of novel sequence. The interspecies introgression landscape revealed the unique genetic diversity and functional variation among the indicine cattle populations. Recently, the Bovine Pangenome Consortium (BPC) was launched (https://bovinepangenome.github.io/, accessed on 1 January 2024) [17]. The BPC aims to develop a community-agreed pangenome reference that will serve as a public online resource for the research community and lay the foundation for future trait-based breeding, bovine genome selection, and adaptive introgression.

## 4. Comprehensive Functional Annotation of the Bovine Genome

To improve genome annotation and understand the biological function of the bovine genome, a series of gene expression atlases covering major tissues and organs have been reported. Using digital gene expression tag sequences, the first Bovine Gene Atlas was generated from three growth stages (fetal, juvenile, and adult) and three cattle cell lines [43] (Table 2). This study systematically explored the relationship among gene expression, gene function, and tissue. With the continuous development of next-generation sequencing (NGS) technologies and bioinformatic algorithms, a high-resolution gene expression atlas was constructed from 135 bovine tissue samples, covering 51 tissue types (heart, brain, muscle, adipose, gland, etc.) [44] (Table 2). This study not only identified 19,356 novel transcripts, but also obtained a series of housekeeping genes (2654), tissue-specific genes (477), and co-expression genes (237) to facilitate a better understanding of the biological function and evolution of multiple tissues in cattle. The characterization of promoters is critical to understanding the patterns that regulate gene expression. Based on the RAMPAGE approach, a promoter activity atlas was generated, and transcription start sites were identified in 31 bovine tissues [45] (Table 2). The comprehensive annotation of the bovine genome in such an extensive collection of tissues contributes to our understanding of gene expression in cattle, reducing the gap in knowledge of transcriptome regulation underlying economically important traits.

With the implementation of FAANG, the functional annotation of genomic regulatory elements is essential for the understanding and efficient use of the genome sequence. The first genome-wide profiling of regulatory elements was established through epigenomic datasets, such as histone modifications, DNA accessibility, and DNA methylation, in bovine rumen epithelial cells [46] (Table 2). Regulatory elements in open chromatin regions were identified through ATAC-seq in liver, muscle, and hypothalamus tissues. The study predicted potential master regulatory factors, namely, HNF4, MEF2, and SOX, in each of the three tissues, and combined transcriptomic data to confirm some candidate target genes [47] (Table 2). The liver-specific regulatory elements (REs) were systematically characterized by integrating multi-omics datasets, including histone modification, chromatin accessibility, gene expression, and functionally involved in liver development and immune processes [48] (Table 2). Meanwhile, the Cattle Genotype-Tissue Expression atlas (CattleGTEx) was constructed and functionally annotated using multi-omics data [19] (Table 2). This study evaluated the tissue-sharing patterns of genetic regulatory effects among 23 distinct tissues. These results further demonstrate the pivotal role of genomic functional annotation in understanding genomic regulation and complex trait variation in cattle.

## 5. Research Focus and Applications of the Bovine Genome

To understand the genetic basis of economic traits of domestic animals, genome research is an essential prerequisite. With the advent of relatively low-cost whole-genome sequencing, it is possible to link cattle phenotypes to variation at the genome level. The 1000 Bull Genomes Project has been launched to accelerate genetic gain in domestic cattle by providing annotated sequence variants and genotypes [15]. Daetwyler et al. [49] completed whole-genome resequencing of 234 bulls from three breeds (Holstein-Friesian, Fleckvieh, and Jersey breed), and identified 28.3 million variants, several of which were functionally associated with milk production (*DGAT1*) and curly coat (*KRT27*) (Table 3). Bouwman et al. [50] conducted a meta-analysis for stature based on imputed whole-genome sequence data from 58,265 cattle among 17 populations, and identified candidate genes (*PLAG1*, *NCAPG–LCORL*) associated with body size (Table 3). Qiu et al. [51] investigated genome variation between wild yak and domestic yak. They detected selection signatures of 209 candidate genes in the domestic yak, several of which were associated with behavior (*ADCYAP1R1* and *SCRIB*), docility (*PLXNB1*), sperm development (*TTLL1* and *RHPN1*), and early pregnancy (*RHOD*). Li et al. [52] examined the genomic diversity, population genetic structure, and selection signatures of 113 samples across nine yak breeds. They identified candidate genes primarily related to milk quality (*OPLAH* and *GRINA*), meat quality (*ZRANB1*), heat stress (*NFAT5*, *HSF1*, and *SLC25A48*), neurodevelopment (*SUSD4*, *INSYN1*, and *PPP1CA*), and disease resistance (*CDK2AP2*, *PLEC*, and *CYB5B*) (Table 3). Zhang et al. [21] constructed a genetic structural variant map using genome resequencing analysis, and identified genes predominantly associated with the nervous system (*MAGI2*), behavior (*MAGI2*, *GAD2*, *GRIK2*), immunity (*NAFT*), and reproduction (*SMOC2*). In buffaloes, Luo et al. [32] detected selective genes correlated to brain development and cognition (*TEAD1*, *OXTR*, *ADYC3*) in swamp buffalo, and identified selective genes related to fecundity (*ESR1*), milk production (*METTL17*, *RNASE2*, *RNASE4*), and body size (*IGF2BP2*) in river buffalo (Table 3). Meanwhile, numerous studies based on reference genomes have identified genetic variations associated with economically important traits in water buffalo. These variations were closely related to heat stress [32,53,54], reproduction [6,55,56], milk production [57,58,59], body coat color [60,61], and disease resistance traits [62,63,64].

The genomic diversity analyses of zebu cattle may provide new insights into the genetic mechanisms by which they adapt to various ecological environments [67]. Kim et al. [65] analyzed patterns of African cattle genetic variation from five indigenous populations. They found the highest genetic diversity and identified important candidate genes related to environmental adaptations, such as circadian rhythm (*HCRTR1*), anemia (*STOM*, *SLC40A1*, *SBDS*, *EPB42*, *RPS26*), coat color (*KIT*, *MITF*, *PDGFRA*, *MC1R*), horn development (*FGF18*), heat tolerance (*SOD1*, *PRLH*), and tick resistance (*BOLA*) (Table 3). Iqbal et al. [66] performed genomic variant analyses among eleven important indicine breeds (e.g., Sahiwal, Red Sindhi, Tharparkar) of Pakistan. Functional annotation identified candidate genes, such as *WNT* and *VSMC*, for adaptation to heat tolerance traits in Pakistani indicine breeds, and *VEGF* and *HIF1* in highland-adapted Pakistani indicine breeds. In gayal, Chen et al. [24] identified expanded gene families (e.g., *MYH*, *DHPR*, *ROCK*), a positive selection gene (*MLCK2*), segmental duplication, and core drive genes (e.g., *RYR2*, *TNNI3*, and *ACTC1*) based on the comparative genome and transcriptome analyses, mainly related to odor sensation and cardiac function (Table 3). Li et al. [36] detected several differentially expressed genes on the newly derived chromosome 2, which is functionally shown to be strongly associated with muscle traits in gayal, an adaptation to the alpine valley they inhabit.

In the post-genomic era, the characterization of regulatory variants that affect gene expression is crucial to unravel the molecular mechanisms underlying economic traits in cattle. Liu et al. [19] performed transcriptome-wide association and colocalization analyses to reveal the regulatory mechanisms between gene expression and 43 economically important traits in cattle. Xiang et al. [68] quantified the contribution of variants using multi-omics data such as gene expression, concentration of metabolites, and histone modification; proposed a Functional-And-Evolutionary Trait Heritability score to rank variants; and provided a set of biological priors for cattle genomic selection worldwide. Subsequently, integrated transcriptome and chromatin accessibility revealed structural variants in the promoters of hypoxia genes (*ARNT*, *GATA1*, *MAFG*, *KLF5*, *HOXB5*) that have potential functions [28]. Single-cell RNA-seq analysis of domestic yak and taurine bovine lung tissues showed that hypoxia genes were explicitly enriched in yak cell clusters, such as *EPAS1* in endothelial cells and *SFTPC* and *SCGB3A2* in epithelial cells [28].

## 6. Advances in the Adaptive Evolution of Bovine

Domesticated cattle originated from wild aurochs (*Bos primigenius*) and became the most crucial farm animal resource, consisting of taurine and indica lines of cattle in the Middle East/Europe and the Indian subcontinent, respectively [69]. Although domesticated cattle have spread worldwide and inhabit diverse environments, the influence of selection pressure has led to unique and vital phenotypes among modern cattle populations [70]. Studies have shown the presence of gene flow from African cattle in European cattle populations, contributing substantial genomic components to the offspring of New World southern European cattle breeds, and identified genes functionally relevant to neurodevelopment (*PHYHIP*), fatty acid metabolism (*FADS2*), and immune function (*FCRL1*) [71,72]. A study on adaptive traits of indica cattle in southern Europe found genomic regions that have had introgression from indicine cattle into white cattle under positive selection, and identified genes with functions related to growth traits (*CPNE4* and *SBF2*), body weight (*SLC25A48*, *CXCL14*, *FBXL21*), body size (*CAMLG*, *DDX46*, *TXNDC15*, *CATSPER3*, *PITX1*), and feed efficiency (*CRISPLD2*, *SERPINB10*, *ZDHHC7*, *KIAA0513*, and *FAM92B*) [73].

In terms of the historical evolution of indigenous cattle breeds in East Asia, Chen et al. [74] found that the East Asian cattle population consisted primarily of three distinct ancestors (East Asian taurine ancestry, Eurasian taurine ancestry, Chinese indicine ancestry) by comparative genomic analysis. This detected adaptive candidate genes mainly related to bitter taste (*T2R12*, *TAS2R9*, and *TAS2R6*), tropical environments (*HSPA1A*, *HSPB8*), and hypoxic environments (*COPS5*, *SDHD*, *IL1A*, *IL1B*, *RYR2*, *MMP3*, and *EGLN1*). Subsequently, the global genetic diversity of indicine cattle was explored, utilizing whole-genome sequencing data from 354 samples across 57 breeds. Genomic analysis has found that indicine cattle may migrate into East Asia along coastal routes rather than inland routes, and identified adaptive candidate genes functionally associated with morphology (*PFN1*, *CAMTA2*, *ENO3*), immune (*SPAG7*, *MST1R*, *MON1A*), and heat tolerance (*LIPH*, *FGF22*, *TRPA1*, *APELA*, *CALB2*, etc.). Structural variation in the East Asian bovine genome plays an essential role in adaptation to local environments. Functional investigations have shown that SVs are linked with genes enriched in environmental adaptation pathways, including epidermal differentiation (*CRNN*, *SBSN*), skin barrier (*SPINK5*), and disease resistance (*SPN*) [75]. In addition, introgression analysis based on SVs of the domestic yak genome separated the different evolutionary origins of domestic yak. It detected potential selection signals from wild yak and introgression genes from cattle, such as *KIT* gene introgression triggering the formation of white yak coat color [76].

## 7. Construction and Application of Bovine Genome Datasets

With exponential development of bovine genomics research, data acquisition, integration, and utilization have brought unparalleled opportunities for interpreting the genetic regulation mechanism underlying the economic traits of cattle. The Bovine Genome Database (BGD) was developed to support genomics research by providing genome annotation and functional information mining tools, such as JBrowse, BovineMine, and BLAST [77]. The Bovine Genome Variation Database (BGVD) has been developed and provides multiple types of variants, including SNPs, indels, and CNVs [16]. Users can quickly retrieve the distribution patterns of these variations through six types of search tools, and further visualize the relationships between variants and genomic selection features. The Exomes Aggregate of Bovine (ExAgBov) was constructed to support annotated variations by predicting open reading frames, UTRs, and splice regions [78]. Meanwhile, a comprehensive Cattle Quantitative Trait Locus Database (Cattle QTLdb) was designed to facilitate users to accomplish many data meta-analyses and comparison tasks in cattle [79]. The current Cattle QTLdb (Release 50, 25 August 2023) contains 193,898 QTLs that represent 680 different base traits and 292 trait variants from 1130 publications. These datasets provide valuable resources to enable the animal resource research community to gain more genomic variants in genomics applications. To accommodate the increasing amount of genomics data, AgAnimalGenomes, a cross-species database, was built to mine the gene features of massive public data and improve the refinement of gene models [80].

IAnimal was developed to present genome visualization and functional annotation by multi-omics data in farm animals and model organisms [81]. Interestingly, based on deep learning models (BioBERT and AutoNER), the retrieval of “genes” and “traits” from literature databases has biological implications for understanding how gene expression regulates traits at the omics level. In addition, an animal metagenome database was designed and developed to collect and integrate the metagenomic sequencing data with host information of multiple animal species. By comparing and analyzing data, users can mine interesting information that helps them understand the ecological basis of microbial communities [82].

## 8. Challenges in the Bovine Genome and Future Perspectives

With the innovation of sequencing technology and assembly algorithms, the bovine genome sequence is gradually improved, which promotes the research of functional genomes. Due to the temporal and spatial specificity of gene expression, a challenge has been raised to elucidate regulatory element functions in the bovine genome. Meanwhile, a series of studies have reported candidate genes for important economic traits of cattle, but few of them can be used in bovine breeding and production. With the iteration of sequencing technology, there is a rapid accumulation of datasets, and various database types, but no unified and authoritative bovine database. Meanwhile, with the rise of modern animal husbandry, many bovine breeds are facing a decline in genetic diversity.

Despite these challenges, the prospects for bovine genomics research include the integration of multi-omics data to mine functional genes for important breeding targets, the application of biological breeding technology to make innovative utilization of candidate genes, the merging and integration of databases to avoid data redundancy and reduce computing power, and the establishment of genetic repositories to prevent the narrowing of the gene pool. Furthermore, genomic research can provide insights into bovine behavior patterns and needs, enabling the development of more welfare-friendly management practices. These advancements hold promise for enhancing our understanding of the genetic regulatory basis of economic traits in cattle and improving breeding programs and management practices in livestock.

## Figures and Tables

**Table 1:** Research progress on bovine genome assembly.

Species Category	Breed	Genomics Version	Sequence Size (Gb)	Contig N50 (Mb)	Scafford N50 (Mb)	References
Cattle	Hereford	UMD *B. taurus* 2.0	2.87	0.08	1.25	[13]
	Hereford	ARS-UCD1.2	2.72	25.9	103.31	[25]
	Simmental	ARS_Simm1.0	2.86	70.8	102	[26]
	Charolais	--	3.2	87	88	[27]
Yak	Domestic yak	*BosGru*_v2.0	2.65	0.02	1.41	[21]
	Domestic yak	*BosGru*3.0	2.83	44.72	114.39	[22]
	Domestic yak	--	2.61	44.91	104.02	[28]
	Wild yak	--	2.83	0.06	16.3	[29]
	Wild yak	--	2.63	38.28	103.9	[22]
Buffalo	Mediterranean	UMD_CASPUR_WB_2.0	2.83	0.022	1.41	[30]
	Bengal buffalo	Bubbub1.0	2.77	0.025	6.96	[31]
	Italian Mediterranean buffalo	UOA_WB_1	2.65	18.8	117.2	[23]
	Fuzhong swamp buffalo	--	2.63	8.8	117.3	[32]
	Murrah river buffalo	--	2.64	3.1	116.1	[32]
	African buffalo	--	2.68	0.043	2.4	[33]
Zebu	Nelore cattle	*B. indicus*_1.0	2.67	0.03	106.31	[20]
Gayal	Drung cattle	--	2.85	0.01	2.74	[34]
	Nagaland	NRC_Mithun_1	3	0.028	1	[35]
	Drung cattle	Drung_v1.2	2.74	0.157	4.08	[24]
	Drung cattle	--	2.57	27.2	--	[36]

**Table 2:** Progress in functional annotation of bovine genomes.

Project	Samples	Results	References
Bovine Digital Gene Atlas	Three growth stages (fetal, juvenile, and adult) and three cattle cell lines	This digital gene expression profile investigates the relationship between gene expression, tissue, and gene function.	[43]
Bovine Gene Expression Atlas	135 bovine tissues in adult beef cattle, covering 51 tissue types	This study identified 19,356 novel transcripts; and detected 2654 HKGs, 477 TSGs, and 237 hub genes.	[44]
A Promoter Activity Atlas	31 bovine tissues	This study identified and characterized transcription start sites, and shortened the gap between genotype and phenotype.	[45]
Bovine Epigenomic Landscape	Rumen	This study established the first global map of regulatory elements (15 chromatin states), and demonstrated the correlation among nutritional elements, chromatin states, gene activities, and phenotype outcomes.	[46]
Open Chromatin Profile	Liver, muscle, and hypothalamus	This study predicted potential master regulatory elements in these three tissues, namely, HNF4, MEF2, and SOX factors, respectively.	[47]
A Ruminant-Specific Regulatory Element Profile	Liver	This study systematically characterized the dynamic functional landscapes, and identified a core set (*n* = 6359) of ruminant-specific REs.	[48]
Cattle Genotype-Tissue Expression Atlas	More than 100 tissues/cell types	This study described the transcriptomic landscape, and evaluated the tissue-sharing patterns of genetic regulatory variants.	[19]

**Table 3:** Functional studies of candidate genes in several bovine species.

Species Category	Trait	Candidate Genes	References
Cattle	immune	*IFNAR2*, *IL23R*, *IL24*, *IL15*, *LEAP2*	[13]
	reproduction and dietary habitats	*TAS2R46*, *QRICH2*, *PRDM9*, and *HSPA1A*	[40]
	milk production	*DGAT1*	[49]
	curly coat	*KRT27*	[49]
	body size	*PLAG1*, *NCAPG*–*LCORL*	[50]
Yak	sensory perception	*GPCR*, *PLXNB1*	[21,51]
	hypoxia response	*ADAM17*, *ARG2*, *ARNT*, *GATA1*, *MAFG*, *KLF5*, *HOXB5*, *SFTPC*, *SCGB3A2*, *EPAS1*	[21,28]
	nutrient metabolism	*CAMK2B*, *GCNT3*, *HSD17B12*, *WHSC1*, and *GLUL*	[21]
	behavior	*MAGI2*, *GAD2*, *GRIK2*, *ADCYAP1R1*, *SCRIB*	[22,51]
	immunity	*NAFT*	[22]
	reproduction	*SMOC2*, *TTLL1*, *RHPN1*, *RHOD*	[22,51]
	disease resistance	*CDK2AP2*, *PLEC*, and *CYB5B*	[4]
	heat stress	*NFAT5*, *HSF1*, and *SLC25A48*	[4]
	milk quality	*OPLAH* and *GRINA*	[4]
	neurodevelopment	*SUSD4*, *INSYN1*, *PPP1CA*, *MAGI2*	[22,52]
	meat quality	*ZRANB1*	[52]
Buffalo	brain development and cognition	*TEAD1*, *OXTR*, *ADYC3*	[32]
	fecundity	*ESR1*	[32]
	milk production	*METTL17*, *RNASE2*, *RNASE4*	[32]
	body size	*IGF2BP2*	[32]
Zebu	heat tolerance	*HSPA4*, *SOD1*, *PRLR*, *WNT*, *VSMC*	[65,66]
	resistance to tick infestation	*BOLA*	[65]
	hypoxia	*VEGF*, *HIF-1*	[66]
Gayal	cardiovascular function	*MYH*, *DHPR*, *ROCK*, *MLCK2*, *RYR2*, *TNNI3*, *ACTC1*	[24]
	muscle traits	*TTN*, *NEB*, *MYH1*, *MYH2*, *MYH4*	[36]
